# Chemical-induced variations in callus regenerated *Atropa acuminata* plants: A study on Ethyl Methanesulfonate effects

**DOI:** 10.1016/j.btre.2025.e00888

**Published:** 2025-03-21

**Authors:** Shabeer Ahmad Dar, Ishfaq Shafi Khan, Gousia Majeed, Sumira Tyub, Irshad Ahmad Nawchoo, Azra Nahaid Kamili

**Affiliations:** a Plant Tissue Culture Laboratory, Centre of Research for Development University of Kashmir, Srinagar, J&K, 190006, India; b Cytogenetics and Molecular Biology Research Laboratory, Center of Research for Development, University of Kashmir, J and K, 190006, India; c Departmet of Environmental Science, University of Kashmir, Srinagar, 190006, J & K, India; d Department of Botany, University of Kashmir Srinagar, J and K, 190006, India; e Department of Botany, Central University of Kashmir, Ganderbal, J and K, India

**Keywords:** Callus, EMS, *In vitro*, Mutant, Thidiazuron, *Atropa acuminata*

## Abstract

*Atropa acuminata* Royle ex Lindl. a member of the solanaceae family is a critically endangered medicinal herb indigenous to the Himalayan region of Kashmir and revered for its therapeutic and economic significance. Ethyl Methanesulfonate (EMS), a chemical mutagen, is commonly used to induce mutations for plant breeding and research purposes. In this study, callus cultures derived from leaf explants of *A. acuminata* were cultivated on Murashige and Skoog (MS) medium supplemented with Thidiazuron (0.1 mg/l) and treated with varying concentrations of EMS (0.1%, 1.0%) over defined time intervals. The impact of EMS concentrations and exposure durations, as well as their interactions, on the characteristics of regenerated callus were rigorously examined. The proliferation of shoots exhibited a diminishing trend with escalating EMS concentrations. Additionally, EMS elicited substantial alterations in the morphological and physiological attributes of the regenerated plants. Among the fifteen traits evaluated, mutant lines designated as M10, M11, and M6 displayed the most pronounced variations in morphological and physiological parameters compared to the control. Notably, the mutant line M10 exhibited significantly elevated shoot dry weight, root fresh weight, chlorophyll, and carotenoid contents demonstrating up to 98%, 31%, 348%, and 642% enhancements respectively as compared to the control. These mutant lines hold promise for further exploitation in augmenting physiological characteristics and enhancing the yield and quality of terpene glycosides in *A. acuminata*.

## Highlights

• The proliferation of shoots exhibited a diminishing trend with escalating EMS concentrations. Additionally, EMS elicited substantial alterations in the morphological and physiological attributes of the regenerated plants.
• In comparison to EMS, leaf explants of *Atropa acuminata* demonstrated a superior response in shooting, rooting, and hardening characteristics
• Among the fifteen traits evaluated, mutant lines designated as M10, M11, and M6 displayed the most pronounced variations in morphological and physiological parameters compared to the control. Notably, the mutant line M10 exhibited significantly elevated shoot dry weight, root fresh weight, chlorophyll, and carotenoid contents, demonstrating up to 98%, 31%, 348%, and 642% enhancements, respectively, compared to the control.
• These mutant lines hold promise for further exploitation in augmenting physiological characteristics and enhancing the yield and quality of terpene glycosides in *A. acuminata*.
• With increase in concentration of EMS, there were reduction in the morphological and physiological characters

## 1 Introduction

Medicinal plants from Kashmir Himalayan such as *Saussurea costus, Atropa acuminata,* and *Podophyllum hexandrum*, have played a crucial role in traditional and modern medicinal practices in the Kashmir Himalayas. Historically, these plants have been integral to Unani, Ayurvedic, and folk medicine, while contemporary research continues to explore their pharmacological potential. They are valued for their potential health benefits and have been used for centuries in traditional medicine practices. The trend towards using medicinal plants is growing globally, driven by factors such as increasing interest in natural remedies, concerns about side effects of synthetic drugs and a desire for sustainable healthcare options [1]. Among countless number of medicinal plants, *A. acuminata*, classified within the Solanaceae family, represents a critically endangered medicinal herb endemic to the Kashmir Himalaya region. Acknowledged for its pharmacological significance and economic value, this species garners attention due to its potential therapeutic applications and socioeconomic relevance [2]. *A. acuminata* is indigenous to the Western Himalayan Mountains, and spans from Kashmir to the adjacent hills of Himachal Pradesh at elevations ranging from 1800 to 3600 meters above sea level [3]. Renowned for its medicinal attributes, the plant harbors bioactive alkaloids of therapeutic importance alongside ethno pharmacological properties. It has historically featured prominently in traditional medicine for its efficacy against ailments such as arthritis, muscle pain, joint disorders, and associated inflammatory conditions [4]. The plant stands as a primary reservoir of tropane alkaloids, notably atropine and scopolamine, renowned for their anticholinergic properties. The natural populations of the plant are characterized by low densities which is a consequence of both excessive harvesting for medicinal purposes and the protracted periods of seed dormancy coupled with seed sterility. In this context, tissue culture not only offers an alternative avenue for the propagation of this critically endangered plant but also facilitates the generation of superior varieties with enhanced secondary metabolite production. For instance, micropropagation and somatic embryogenesis techniques in *Podophyllum hexandrum* have led to increased podophyllotoxin accumulation compared to wild populations [5]. Similarly, *in vitro* strategies in *Withania somnifera* have successfully enhanced withanolide content, demonstrating the potential of tissue culture for optimizing bioactive compound yield [6]

Mutation serves as a pivotal tool for elucidating the molecular characteristics and functionalities of genes. In the realm of plant biology, mutations broaden the spectrum of genetic diversity *in vitro*, thus providing a foundation for breeding endeavors [7]. Under these conditions mutagenic treatments are meticulously tailored and screening methodologies for mutant populations are refined to achieve specific modifications [8]. EMS is a chemical mutagen with notable efficacy in inducing mutations. EMS is one of the most common used alkylating agents that can induce chemical modification of nucleotides through the introducing active alkyl group, which creates base changes and nucleotide mutations [[9], [10], [11]]. Modifications caused by alkylating agents include the N_7_ and O_6_ of guanine (G), N_3_ and N_7_ of adenine (A), N_3_, O_2_, and O_4_ of thymine (T), N_3_, O_2_, and N_4_ of cytosine (C), and the phosphonate backbone (Fu et al., 2012). The EMS primarily induces the changes in guanine (Boysen et al., 2009). .EMS is a type of non-transgenic chemical mutagen, and EMS mutagenesis is an important way to obtain mutations and the discovery of new genes for plants [12]. In the current study EMS has been selected to study its impact on the generation and enhancement of physiological traits in *A. acuminata* with the overarching objective of generating novel mutant lines of this plant species.

## 2 Materials and methods

### 2.1 Plant material and callus induction

The seeds obtained from wild population of *A. acuminata* were utilized as the initial material for this study. Acquisition of the seeds was carried out at the Kashmir University Botanical Garden, located in Srinagar, Jammu and Kashmir, India. The collection of the plants used in this study complies with local or national guidelines with no need for further affirmation. However, under specimen number 2862—KASH herbarium a voucher sample was submitted in the Centre of Biodiversity, Department of Botany, University of Kashmir.

Subsequently, the seeds were germinated under controlled laboratory conditions to raise seedlings and generate explants essential for the experimental procedures conducted in this study.

#### 2.1.1 Sterilization of seeds

Prior to germination the seeds undergo a thorough cleaning process utilizing a commercial laboratory-grade detergent (Labolene) in combination with a surfactant (Tween 20). Following general washing with tap water to remove any residual contaminants, the seeds given chemical sterilization using a 0.2% solution of mercuric chloride (HgCl_2_) for 06 minutes. HgCl₂ was used due to its proven efficacy in eliminating contaminants, as compared to other surface sterilization agents. Proper washing steps ensured minimal residue.

#### 2.1.2 Media used

The culture medium employed in this study is Murashige & Skoog's formulation [13], supplemented with 3% sucrose and adjusted to a pH range of 5.6–5.8. Agar at a concentration of 0.8% was utilized for solidification of the medium. Sterilization of the culture medium was achieved through autoclaving at 121⁰C for 20 minutes. Depending on the stage of micro propagation and the nature of the explants, the medium composition varied between basal formulations and those supplemented with growth hormones. The selection of this medium is justified by its ability to support robust shoot proliferation, efficient root induction, and enhanced secondary metabolite content, which are crucial for the *in vitro* regeneration of *Atropa acuminata*. Previous studies have demonstrated that this medium provides an optimal balance of macronutrients, micronutrients, and growth regulators, making it superior to other formulations such as White's medium for sustaining long-term culture and maximizing the yield of bioactive compounds [5,6]

#### 2.1.3 Seed germination

Seed germination was initiated on Murashige and Skoog (MS) basal medium. The seeds were aseptically inoculated onto autoclaved media under laminar airflow. Following inoculation, the culture vials containing the seedlings were maintained in culture room. These *in vitro*-raised seedlings served as the source material for explants used in callus induction and shoot multiplication. The seedlings were dissected into small segments to obtain the desired explants.

#### 2.1.4 Induction of mutation under *in vitro* conditions

The preparation of explants commenced by excising nodal segments from *in vitro* propagated plantlets. These explants were subsequently cultured on hormone-free Murashige and Skoog (MS) basal medium supplemented with 3% sucrose and 0.8% agar, adjusted to a pH of 5.8. The medium after dispensing in suitable culture vials was autoclaved for 20–25 min at a pressure of 15 lb and at a temperature of 121 ^°^C. The cultures were maintained at 23± 5 °C with 55–65% RH and exposed to 16 hr light period using cool fluorescent (3000 lux) tubes.

#### 2.1.5 Callus induction

Leaf explants were excised from 4-week-old *in vitro*-cultured seedlings and transferred to Murashige and Skoog (MS) medium supplemented with 0.1mg/L thidiazuron (TDZ). Subsequent to transplantation, explants were sub cultured onto fresh medium of identical composition at five-week intervals. Following these sub culturing events, callus initiation was observed. The resulting callus tissue was sectioned into small fragments to undergo EMS treatment.

### 2.2 Ethyl Methanesulfonate (EMS) application

One percent stock solution of EMS was prepared and subsequently diluted to obtain working solutions at final concentrations of 0.1%, 0.2%, and 0.3%, denoted as E1, E2, and E3, respectively. Prior to application the EMS solutions was sterilized. Callus cultures established on growth regulator-free Murashige and Skoog (MS) medium for one week were immersed in the EMS solutions at the aforementioned concentrations. The treatments were carried out on a rotary shaker set at a constant speed of 145 rpm at room temperature. The callus cultures without EMS treatment worked as control.

At this stage, every plant regenerated from EMS-treated callus was designated as a mutational event, leading to the creation of a cloned population of identical age for each plant. From each treatment group, regenerated plants demonstrating favorable growth traits were chosen, and individual codes were assigned to each plantlet regenerated from callus. Meanwhile, plants originating from control callus were carefully examined. By culturing subsequent nodes, a clone was produced for each distinct code. After an acclimatization period, the morphological and physiological characteristics of seedlings within each clone were assessed.

### 2.3 Acclimatization

Plantlets with a proficiently established root system were taken for acclimatization in *ex vitro* conditions. During the acclimatization process plantlets exhibiting well-developed shoot and root systems were taken out of flasks. The basal region of the plantlets was cleansed with sterile water to eliminate any remnants of adhering medium. Subsequently, the cleaned plantlets were transplanted into small pots containing autoclaved soil in the greenhouse, maintained at a regulated temperature of 20±4 °C and relative humidity of 65%. These plants were watered regularly. The observed survival rate of the plantlets was recorded as 77%.

This experiment was executed utilizing a completely randomized design comprising three replications. The collected data underwent statistical analysis utilizing SAS software, followed by a comparison of means using Duncan's multiple range tests at a significance level of 1%, facilitated by MSTAT-C software.

## 3 Analysis of morphological traits and biochemical parameters

### 3.1 Morphological traits

The plants derived from both control and EMS treated calli were examined for several morphological characteristics including plant height, leaf surface area, and stem diameter. Furthermore, measurements of fresh and dry weight of plantlets/shoots were recorded. Plants of similar age were harvested for this investigation, and various measurements and weights were documented. The dry weights of the shoots were determined after incubation in an oven at 40 °C for a period of 24 hours. The Digitizer software was employed to compute the surface area of the leaves.

### 3.2 Determination of total anthocyanins level

The anthocyanin content of each extract was quantified using the methodology outlined by Mita et al. [14], as described by the following equation:

$$A=A_{0530}-\left(0.25*A_{657}\right)$$

Where, A is light absorption (the subscripts and superscripts represent wavelengths at which light absorption was read).

### 3.3 Carbohydrate measurement

The total carbohydrate content of fully matured leaves was determined in accordance with the methodology outlined by Ganai *et al*. [15]. Initially, 0.1 g of fresh leaf or callus material was immersed in 10 ml of alcohol and incubated at 60 °C for 1 hour in an incubator. The resultant extract was then decanted into a 25 ml volumetric flask, and the residue underwent a secondary extraction. Alcohol was added to the flask to achieve a final volume of 25ml. Subsequently, the entire sample was subjected to evaporation in a water bath to remove the alcohol leaving behind a residue that was dissolved in 1.0 ml of 5% phenol solution mixed with 1 ml of distilled water. Following this, 5 ml of analytical grade sulfuric acid was added and thoroughly mixed with a glass rod through vertical agitation until a brownish-red complex formed. The absorbance of the resulting solution was measured at 490nm using a double beam UV–Vis spectrophotometer. The corresponding concentration was determined by referencing a standard curve prepared using a glucose solution.

### 3.4 Soluble protein measurement

The protein content was quantified utilizing the method described by Bradford [16]. Initially, 0.1 g of fresh leaf or callus was homogenized in 1.5ml of extraction mixture using a mortar and pestle. The resulting homogenate was transferred to 2.5ml centrifuge tubes and centrifuged at 10,000 rpm at 4°C for 10 minutes. Subsequently, 0.5ml of the extract aliquot was utilized for protein content determination. An equivalent volume of chilled 10% trichloroaceticacid (TCA) was added to the 0.5ml aliquot, followed by centrifugation at 4000 rpm for 10 minutes. The supernatant was discarded, and the remaining pellet was washed with acetone. The pellet was then dissolved in 0.5ml of 0.1N sodium hydroxide (NaOH), and the final volume was adjusted to 1ml by adding double-distilled water (DDW). Five (5ml) of Bradford's reagent were added to 0.1ml of the aliquot and vortexed. The tubes were allowed to stand for 10 minutes to facilitate optimal color development. Subsequently, the absorbance was measured at 595nm using a double beam UV–Vis spectrophotometer. The soluble protein concentrations were quantified using a standard curve prepared from Bovine Serum Albumin (BSA) standard from Sigma, USA. The protein content was expressed mg g-1fw fresh weight.

## 4 Results

**Shoot and root regeneration response after EMS treatment under in vitro conditions**

### 4.1 Treatment of callus

The obtained callus was subjected to treatment with different concentrations of EMS ranging from 0.01% to 0.1% for a duration of 1 hour, prior to being cultured on regeneration media consisting of thidiazuron (TDZ) at a concentration of 12μM in combination with indole-3-butyric acid (IBA) at 3.5μM for shoot multiplication. For rooting, the callus was cultured on Murashige and Skoog (MS) medium supplemented with IBA at a concentration of 8μM. These particular concentrations of media components were selected based on their previously observed enhanced growth response under standard growth conditions.

**Fig. 1:**
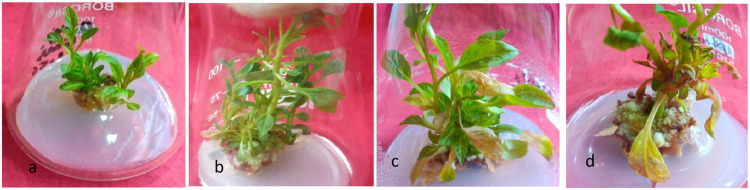
Effect of EMS on shoots of *in vitro* raised microshoots from callus explants of *A. acuminata*. a: Control. b: 0.03% EMS. c: 0.05% EMS. d: 0.9 % EMS.

In the initial experimental trial, EMS exhibited a notable influence on the potential for shoot regeneration, resulting in an increase in shoot numbers. Notably, there was an upward trend in shoot numbers observed up to 0.05% EMS concentration (14.4±0.42), compared to the control group (13.3±0.40). However, the maximum average shoot number was recorded at 0.03% EMS concentration (21.5±0.66). Subsequently, with further increase in EMS concentration, a significant decline in the number of shoots was observed. Concurrently, the length of the shoots exhibited a gradual reduction with increasing EMS concentration, ranging from 3.8±0.66 at 0.01% EMS to 1.2±0.07 at 0.1% EMS (Table 1, Fig. 1, a-d).

**Table 1:** Effect of EMS on regeneration of shoots and roots from *in vitro* raised callus of *A. acuminata*.

	*Average Shoot Number± SD	*Average Root Number± SD	Shoot (%)	Rooting (%)	*Average Shoot length ± SD (cm)	*Average Root length ± SD (cm)	*Total length ± SD (cm) (Root + Shoot)
Control	13.3±0.40^e^	7.6±0.41^e^	100	100	4.1±0.17^e^	4.0±0.11^d^	8.3±0.32^f^
1 Hour	16.1±0.16^ef^	9.2±0.10^e^	90	100	3.8±0.66^d^	3.9±0.31^c^	7.9±0.18^e^
	19.5±0.54^g^	11.0±0.13^f^	100	100	3.8±0.46^d^	3.9±0.42^c^	7.7±0.25^e^
	21.5±0.66^h^	12.6±0.45^h^	100	100	3.8±0.62^d^	3.9±0.30^c^	7.6±0.10^e^
	19.6±0.61^g^	11.4±0.31^g^	100	100	3.6±0.70^d^	3.6±0.50^c^	7.3±0.45^e^
	14.4±0.42^e^	10.3±0.33^f^	100	100	3.5±0.22^bc^	3.6±0.32^c^	7.1±0.25^e^
	12.9±0.13^a^	8.8±0.14^e^	100	100	3.2±0.44^bc^	3.±0.22^c^	6.9±0.25^d^
	11.4±0.24^d^	7.6±0.12^d^	90	100	3.1±0.45^c^	3.3±0.55^c^	6.4±0.13^d^
	11.1±0.11^d^	7.5±0.52^d^	90	100	3.0±0.19 ^c^	3.3±0.15^c^	6.3±0.17^d^
	4.4±0.57^b^	3.0±0.46^b^	75	85	1.4±0.20^a^	2.0±0.15^b^	4.0±0.31^e^
	2.1±0.43^a^	1.0±0.17^a^	70	75	1.2±0.07^a^	1.1±0.21^a^	2.6±0.11^a^

⁎ Values are represented as mean ± SD (n=20), Data was analyzed by ANOVA using Duncan's multiple range test (SPSS 27.0); the values with different superscript along the columns are statically significant at P<0.05. Data scored after 6 weeks of culture period.

**Fig. 2:**
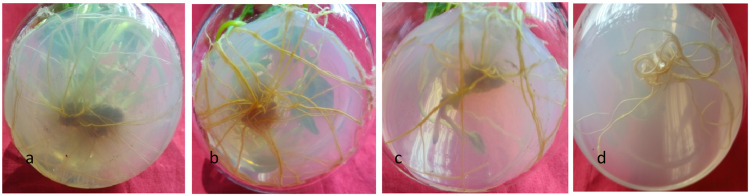
Effect of EMS on rooting of *in vitro* raised microshoots from callus explants of *A. acuminata*. a: Control. b: 0.03% EMS. c: 0.05% EMS. d: 0.9 % EMS.

In the subsequent trial, the influence of EMS on rooting was also examined. EMS exhibited a significant effect on the capacity for root regeneration, with an observed increase in root number up to 0.06% EMS concentration (8.8±0.14), in comparison to the control group (7.6±0.41). Moreover, the highest average root number was recorded at 0.03% EMS concentration (12.6±0.45). However, with further increase in EMS concentration, a decline in root number was noted. Additionally, root length exhibited a gradual decrease with increasing EMS concentration, ranging from 4.0±0.11 in the control group to 1.1±0.21 at 0.1% EMS concentration. Notably, the thickness of adventitious roots was consistently observed to be higher across all EMS concentrations compared to the control group (Table 2, Fig. 2, a-d).

**Table 2:** Comparison of mean some traits related to regeneration from callus treated with EMS.

**Treatment**		Regeneration*(%)	Shoot(No)*	Day (No)*
Concentration	Time			
E0 (control)	T0	92.00^a^	16^a^	15ab
E1	T1	56.77^a^	8ab	32.26^ab^
E1	T2	24.72^abc^	5bc	37.55^ab^
E2	T1	34.30^ab^	4bc	42^a^
E2	T2	0.00^c^	0d	0b
E3	T1	5.88^bc^	1cd	53.57^a^
E3	T2	0c	0d	0^b^

⁎ The letters in the table likely represent statistical groupings based on a post-hoc test, such as Duncan's Multiple Range Test. Same letter means the values are not significantly different from each other.

The dataset was subjected to analysis, employing treatments encompassing a spectrum of EMS concentrations (0.1%, 0.2%, 0.3%, 0.4%, and 0.5%). The investigation elucidated that the diverse EMS concentrations (0.1%, 0.2%, 0.3%, 0.4%, and 0.5%) induced alterations in the frequency and quantity of shoots regenerated from the calli (P<0.01). Notably, the control group exhibited the highest regeneration frequency. Furthermore, the analysis of variance (ANOVA) outcomes underscored a significant (P<0.01) effect of EMS dosage on various attributes of the plants regenerated from treated calli (Table 2).

#### 4.1.1 Hardening

**Fig. 3:**
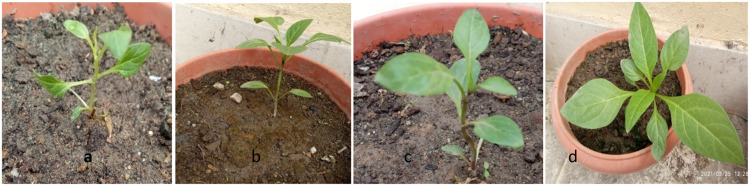
EMS treated *In vitro* regenerated plantlets in green house.

The hardening phase was conducted within a greenhouse characterized by 70% shading and 80% humidity levels. Initially, fifty (50) rooted plantlets originating from mutagen-treated (EMS) sources and an equivalent number of plantlets from the control group were transplanted sequentially from the greenhouse to a net house, and ultimately to field conditions until reaching maturity. Analysis of the gathered data revealed a noteworthy decrease in the percentage survival of plants following their transfer to the greenhouse environment. Notably, plantlets from the control group exhibited a higher percentage survival rate (80%). Conversely, as EMS dosage increased, a significant reduction in percentage survival was observed. The lowest survival rate, at 55%, was recorded among plantlets regenerated from calli treated with 0.1% EMS, as compared to the control group (Table 3, Fig 3).

**Table 3:** Response of EMS treated plants transferred to field conditions in M1 generation of *A. acuminata*.

**Treatments EMS (%v/v)**	**Number of plantlets transferred to field**	**% Survival**
	*CRP	*CRP
Control	21	80
0.01	18	70
0.02	18	70
0.03	17	70
0.04	12	65
0.05	12	60
0.06	13	60
0.07	12	55
0.08	11	50
0.09	10	45
0.1	09	40

Data scored after 4 months (n=20), n=number of plants/treatment*CRP (Callus regenerated plantlets)

### 4.2 Effect of Ethyl Methanesulfonate under *in vivo* conditions

In order to investigate the impact of EMS on the M1 generation via morphological traits, a sample of 10 plants was randomly chosen from among the plants regenerated from callus explants subjected to treatments ranging from 0.01% EMS to 0.08% EMS. Surviving plants were selected from treatments involving 0.09% EMS and 0.1% EMS.

In the M1 population, EMS manifested its influence on plant height, the number of primary branches per plant and seed yield in comparison to the control group. Notably, there was a consistent decline observed in plant height with increasing concentrations of EMS across all treatments. However, a stimulatory effect on the number of primary branches per plant and seed yield was noted at lower concentrations of the mutagen. Specifically, an enhancement in seed yield and the number of primary branches per plant was observed relative to the control at the concentration of 0.03% EMS, recorded as 5.5g per plant and (4.4±0.31), respectively (Table 4).

**Table 4:** Phenotypic variations due to EMS in M1 generation of *A. acuminata* in field conditions.

Treatments EMS (% v/v)	*Average Plant Height ±SD (cm)	*Number of primary branches/plant ±SD	*Average seed number/plant ±SD
	*CRP	*CRP	*CRP
Control	24.4±0.11^d^	3.3±0.12^a^	4.7±0.42^b^
0.01	25.5±0.13^c^	3.8±0.36^a^	4.8±0.42^bc^
0.02	26.0±0.14^c^	4.0±0.43^b^	5.1±0.32^c^
0.03	28.3±0.27^c^	4.4±0.31^b^	5.5±0.10^dc^
0.04	28.7±0.32^b^	4.3±0.52^b^	5.4±0.01^d^
0.05	27.1±0.21^b^	4.0±0.81^b^	5.2±0.14^c^
0.06	26.6±0.23^b^	3.8±0.13^b^	5.0±0.15^c^
0.07	26.3±0.14^b^	3.3±0.15^a^	4.4±0.17^bc^
0.08	25.1±0.13^a^	3.1±0.33^a^	4.2±0.18^b^
0.09	24.4±0.10^a^	3.0±0.11^a^	4.0±0.19^b^
0.1	22.2±0.33^a^	2.4±0.23^a^	3.7±0.15^a^

⁎ Values are represented as mean ± SD (n=10), Data was analyzed by ANOVA using Duncan's multiple range test (SPSS 27.0); the values with different superscript along the columns are statically significant at P<0.05. CRP (Callus regenerated plantlets)

Upon examination of morphological traits, it was observed that the fresh weight and dry weight of plantlets exhibited variations among certain mutants compared to the control counterparts. Specifically, the fresh weights of shoots were notably elevated by 33.3%, 29.2%, and 24.3% in mutants M12, M5, and M10, respectively, relative to the control. Furthermore, the dry weights of shoots in mutants M10, M11, and M6 exhibited increments of +98%, +68.6%, and +44.5%, respectively, compared to the control. Similarly, several mutants displayed increases in both fresh and dry weights compared to the control. Mutants M10 and M6 displayed the highest fresh weights of roots, measuring 4.56 and 4.12 grams, respectively, in contrast to the control (3.55 grams), while mutants M7, M2, and M3 exhibited the lowest fresh weights at 1.19, 1.60, and 1.74 grams, respectively (Table 5). Only four of the assessed mutants displayed higher root dry weights than the control (which measured 1.33 grams), with mutants M6 and M2 exhibiting the highest and lowest root dry weights of 1.47 and 0.24 grams respectively.

**Table 5:** ANOVA of morphological traits in plantlets regenerated from callus of *A. acuminata*.

Source of Variations	Degree of freedom	Shoot fresh weight (g)	Shoot dry weight (g)	Root fresh weight (g)	Root dry weight (g)	Height (cm)	Leaf area (cm^2^)
Treatment	16	13.88**	4.041*	3.21**	0.524**	30.91*	5446.43**
Error	36	0.156	0.002	0.006	0.0016	1.601	32.51
C.V (%)		5.52	1.95	2.17	4.70	5.70	2.09

**^,^*Significant at the 1%and 5%probabilitylevels respectively

The examination of plant height in the obtained mutants revealed an increase in certain cases, syemmetring the trends observed in fresh and dry weights (Table 6). Furthermore, concerning leaf surface area and stem diameter, select mutants displayed an augmentation in these parameters relative to the control group (Table 7).

**Table 6:** Comparison of mean of morphological traits in plantlets regenerated from callus treated with EMS.

Treatment	Code	Shoot fresh Weight (g)	Change (%)	Shoot dry Weight (g)	Change (%)	Root fresh Weight (g)	Change (%)	Root dry Weight (g)	Change (%)
E0T0	M1	6.11d	-	1.79g	-	3.55d	-	1.33c	-
E1T1	M2	4.21^fg^	-43.2	1.22^j^	-30.8	1.60^k^	-54.6	0.24^hi^	-70.80
E1T1	M3	4.06^f^	-38.8	1.01^k^	-42.3	1.74^k^	-50.7	0.26^hi^	-69.62
E1T1	M4	7.50^d^	-3.90	1.58^h^	-8.52	3.84^cd^	+5.55	1.06^d^	-15.43
E1T1	M5	8.17^a^	+29.2	2.41^e^	+28.1	3.94^c^	+8.34	1.20^c^	-0.91
E1T1	M6	7.14^c^	+11.7	2.58^d^	+44.5	4.12^b^	+22	1.47^a^	+15.22
E1T1	M7	4.51^e^	-25.9	1.07^k^	-42.7	1.19^i^	-34.3	0.20^hi^	-80.31
E1T1	M8	5.95^d^	-3.98	2.02^f^	+8.82	2.51^fg^	-25.7	0.52^f^	-47.32
E2T1	M9	9.43^gh^	-50.6	0.60^l^	-69.2	2.38^gh^	-31.2	0.51^f^	-44.49
E1T2	M10	8.02^ab^	+24.3	4.14^a^	+98	4.56^a^	+31	1.28^ab^	+11.01
E1T2	M11	7.95^bc^	+16.6	2.94^c^	+68.6	3.36^d^	-3.01	1.25^b^	+9.47

The letters in the table likely represent statistical groupings based on a post-hoc test, such as Duncan's Multiple Range Test. Same letter means the values are not significantly different from each other.

**Table 7:** Means comparison of morphological traits in seedlings regenerated from EMS treated callus.

Treatment	Code	Height (cm)	Change (%)	Leaf area (cm^3^)	Change (%)	Stem diameter (mm)	Change (%)
E0T0	Control	21.4^de^	-	151.8^h^	-	1.57^g^	-
E1T1	M2	24.3^bcd^	+5.76	140.7^i^	-7.93	2.16^ab^	+41.47
E1T1	M3	21fg	-9.53	169.6^g^	+11.5	2.58^a^	+52.32
E1T1	M4	22cde	+2.90	169^g^	+13.7	2.51^a^	+51.73
E1T1	M5	20.5^ef^	-4.16	220.5^d^	+45.9	2.05^bcd^	+31.14
E1T1	M6	15.5^h^	-20.48	236^c^	+53.2	1.54^fg^	+6.34
E1T1	M7	18.7^fg^	-10.87	181.2^fg^	+16.8	2.10^bc^	+33.49
E1T1	M8	19gh	-18.42	242^bc^	+54.8	1.65^efg^	+7.35
E1T1	M9	13.5^i^	-35.15	126.5^j^	-14.6	1.67^efg^	+10.39
E1T2	M10	21.6^cde^	+1.39	221.7^d^	+41.3	2.34^ab^	+42.50
E1T2	M11	21.5^cde^	+1.56	241.7^b^	+58.4	1.41^efg^	+8.70
E1T2	M12	20.2^def^	-3.00	284.2^a^	+90.1	1.83^cde^	+22.9
E1T2	M13	24.4^ab^	+14.34	144^ij^	-8.58	1.67^cde^	+22.91
E1T2	M14	20.7^def^	-2.70	144.8^hi^	-6.10	1.67^fg^	+5.04
E1T2	M15	16.4^h^	-21.36	210.3^e^	+32.1	1.75^efg^	+12.85

Means in each column followed by a similar letter are not significantly different at the1% level of probability.

The letters in the table likely represent statistical groupings based on a post-hoc test, such as Duncan's Multiple Range Test. Same letter means the values are not significantly different from each other.

### 4.3 Physiological traits

The current investigation unveiled that EMS exerts a notable impact on physiological traits, including soluble protein, carotenoids, and anthocyanins. Upon subjecting the data to analysis of variance (ANOVA), it became apparent that EMS exhibited significant effects on the levels of soluble protein, carbohydrate, and anthocyanins at the 1% significance level (Table 8).

**Table 8:** ANOVA of physiological traits in plantlets regenerated from callus treated with EMS.

Source of variation	Df	Chlorophyll	Carotenoid	Anthocyanins
Treatment	16	0.1609**	0.034**	0.013**
Error	36	0.00007	0.00003	0.00002
C.V		2.286	4.44	3.52

*Significant at the1% levels of probability

The findings revealed that mutant M10 exhibited a notably higher chlorophyll content compared to the control group. Additionally, mutants M6, M11, and M13 displayed the highest chlorophyll contents, measuring 1.06, 1.02, and 1.01 mg/g, respectively surpassing the control level of 0.151 mg/g. Conversely mutants M9 and M8 exhibited the lowest chlorophyll contents with values of 0.267 and 0.438 mg/g respectively (Table 9).

**Table 9:** Comparison of mean of physiological traits in the regenerated plantlets of calluses exposure at EMS.

Treatment	Code	Chlorophyll (mg/g)	Change %	Carotenoid (mg/g)	Change %	Anthocyanin (mg/g)	Change %
E0T0	Control	0.151^m^	-	0.041^n^	-	0.064^m^	-
E1T1	M2	0.297^n^	-25.2	0.0111^n^	-74	0.171^e^	+168.14
E1T1	M3	0.512^j^	+46.8	0.031^m^	-42	0.077^l^	+28.75
E1T1	M4	0.721^i^	+93.5	0.174^f^	+230	0.182^d^	+181.67
E1T1	M5	0.452^e^	+165.1	0.432^f^	+166	0.258^b^	+304
E1T1	M6	1.06^d^	+230	0.344^b^	+580	0.131^h^	+104.89
E1T1	M7	0.96^g^	+131	0.085^j^	+86	0.111^jk^	+68.14
E1T1	M8	0.438^l^	+2.53	0.225^d^	+359	0.110^jk^	+64.77
E1T1	M9	0.267°	-30.5	0.050^l^	+3	0.121^i^	+87.07
E1T2	M10	1.5^b^	+348	0.328^a^	+642	0.154^g^	+130.74
E1T2	M11	1.02^a^	+267	0.104^i^	+120	0.115^ij^	+75.11

Mean in each column, followed by a similar letter are not significantly differentat1% level of probability

The letters in the table likely represent statistical groupings based on a post-hoc test, such as Duncan's Multiple Range Test. Same letter means the values are not significantly different from each other.

Moreover, mutants M5, M6, and M10 showcased the highest concentrations of carotenoids, measuring 0.432, 0.344 and 0.328 mg/g, respectively in contrast to the control value of 0.041 mg/g. Conversely, mutant M2 exhibited the lowest total carotenoids content recorded at 0.011 mg/g. notably all mutants tested displayed higher anthocyanin content compared to the control (Table 9).

## 5 Discussion

EMS is a widely utilized chemical agent for inducing mutations in plants. It induces random point mutations at a high frequency potentially leading to the emergence of genetic diversity within plant populations particularly when minimal variability exists for a specific trait thereby facilitating improvement through mutation breeding (Talebi *et al*., 2012; [9,17,18]). Espina *et al*. [19] have observed that optimizing the effectiveness and efficiency of mutagens while ensuring a high mutation frequency without compromising the generation of desired traits remains crucial. The optimal dosage of EMS varies across different crops with values typically below 1% for rice, soybean and tomato (Talebi *et al*., 2012; Arisha *et al*., 2015; [20]) but potentially higher (up to 1.5%) for certain pepper cultivars [[17], [21],^22^].

In this backdrop present study has been taken to study the influence of EMS concentration on the regeneration potential, as well as other morphological and physiological parameters of regenerated plantlets. In consistence with finding by Zhu *et al*. [23] in the study it was observed that the favorable effects of mutagenesis were weak at higher concentrations. Furthermore, in the current investigation both shoot length and root length exhibited a gradual decrease with increasing EMS concentration. These results are in agreement with the findings of Luan *et al*. [24] in *Ipomoea batatas* and Svetleva and Crino [25] in common beans who have also showed that induced mutations in callus explants resulted in inhibition of growth and development at higher concentrations.

The enhancement induced by the utilization of EMS in *A. acuminata* is evidenced by the observed morphological alterations in the M1 generation. These modifications in growth parameters like plant height, root length, primary branch count, and seed yield within the M1 generation may stem from physiological influences or mutations occurring in structural genes. Similar observations were made by Sikder et al. [20] and Jahan et al. [26], who reported that meiotic irregularities reduced seed germination rates by 20–30%, decreased seedling height by 15–25%, and impaired pollen fertility by 25–40% in *Lycopersicon esculentum* and *Hydrangea macrophylla* during the M1 generation. The observed trends in the M1 generation underscore a clear inverse relationship between EMS concentration and various critical morphological parameters. As EMS concentration increases, there is a notable decline in survival and germination percentages as well as reductions in plant height and root length. Additionally, higher concentrations of EMS manifest in pronounced morphological alterations, including changes in flower and leaf coloration, browning, and leaf curling. These findings are in line with previous research attributing such alterations to a myriad of factors, including cytogenetic damage, physiological disruptions, and imbalances in growth regulator and promoter inhibitors. The consistency of our results with those reported in studies by Aslam *et al*. [27] on *capsicum annuum* and [28] on tomatoes further validates the impact of EMS concentration on plant survival rates. Moreover, our findings align with documented observations of similar alterations induced by EMS in other plant species such as *Coixlacryma-jobi* (Ya, 1996). This convergence of results underscores the broader implications of EMS concentration on plant morphology across diverse plant species and highlights the importance of careful consideration in its application for genetic studies and breeding programs.

The reduction plant height and root length by mutagenic treatments may be due to the uptake and subsequent impact of mutagens on meristematic tissues and germ cells as previously observed by Serrat *et al*. [29]. Such decreases in plant height and root length have also been previously documented in various crop plants by the works of Uma and Salimath [30]. Future enhancement in *Atropa acuminata* could focus on increasing atropine and scopolamine yield, improving *in vitro* shoot proliferation and rooting efficiency, enhancing stress tolerance (*e.g.*, drought and salinity resistance) and optimizing genetic stability for large-scale propagation. Additionally, traits such as faster germination, higher biomass production and resistance to microbial pathogens could be targeted to improve both conservation and commercial viability.

Our results align with those of Talebi *et al*. (2012), who noted a proportional decrease in root length with increasing EMS concentration in *Oryza sativa* L. Furthermore, in *Asteracantha longifolia*, EMS-induced mutagenesis has been shown to exert significant effects on various morphological aspects including plant height, internodes length and leaf size as demonstrated by Behera *et al*. [31]. Consistently, our findings also reveal similar impacts on plant morphology. Similarly, investigations in *Jatrophacurcas* by various researchers, including Kumar and Selvaraj [32] have highlighted significant reductions in root and shoot lengths as well as seedling vigor, particularly at higher concentrations of gamma rays and EMS. Furthermore, the decrease in root length attributed to EMS treatment has been corroborated by Sahi and Ehsanpour [33]. In a study conducted by Kaul and Bhan [34], three rice cultivars were subjected to various mutagenic agents including EMS, DES, and Gamma rays, either individually or in combinations. Their investigation revealed an increase in chlorophyll content. The study is particularly significant as it focuses on a critically endangered medicinal plant species known to yield important compounds such as atropine and scopolamine which are in increasing demand due to consumer interest in natural products. The primary objective of our research was to induce morphological changes in *A. acuminata* so that enhanced content of secondary metabolites is obtained. The utilization of EMS has proven beneficial in increasing the number of regenerated plantlets thus facilitating the increased production of improved *in vitro* raised plants. This may prove useful for conservation efforts by meeting the growing demand of the plant. Future studies should focus on refining the optimization of growth regulators and elicitors to further enhance atropine and scopolamine production in *Atropa acuminata*. Additionally, metabolic profiling and transcriptomic analysis could be employed to understand gene expression changes under different treatments. Testing these refinements would involve comparative *in vitro* and *in vivo* assays, evaluating secondary metabolite content using HPLC/GC–MS and assessing genetic stability through molecular markers

The beneficial alterations obtained in terms of increase in anthocyanin and carotenoid content and favorable changes in morphological trait should promise for augmenting material availability for pharmaceutical extraction while concurrently alleviating pressure on natural habitats. It is worth noting that the type of mutations induced by a specific mutagen is anticipated to remain consistent irrespective of whether it is administered *in vivo* or *in vitro*, as elucidated by Dewhurst *et al*. (2021). Consequently, the mutations obtained through EMS treatment can also be extrapolated to field-grown plants. Numerous researchers have successfully obtained mutants with desirable traits in medicinal plants contributing significantly to the field. These advancements underscore the potential for leveraging mutagenesis techniques to enhance the quality and quantity of medicinal plant-derived products, ultimately benefiting both pharmaceutical industries and environmental conservation efforts.

## 6 Conclusion

the present study highlight the significant impact of EMS treatment on the traits of regenerated plants from callus cultures. A discernible influence of EMS concentration and exposure duration on various plant characteristics has been observed. Notably, lower concentrations of EMS prompted an increase in shoot production, whereas higher concentrations correlated with a decrease in shoot formation. Similarly, shorter exposure durations to EMS exhibited more favorable effects on plant traits compared to prolonged exposure periods. The objective of attaining regenerated plants with enhanced traits, have been accomplished successfully to a considerable extent. These results highlight the potential of EMS treatment as a tool for targeted trait improvement in plant regeneration programs. Further exploration of optimal EMS concentrations and exposure durations could yield even more refined outcomes, paving the way for advanced strategies in plant breeding and genetic enhancement.

## Declarations

### Ethics statement

Not applicable.

### Consent for publication

This work has not been published before, and is not under consideration for publication elsewhere and all authors have approved this work for publication.

### Ethics approval and consent to participate

The plant material used in the present study was collected from Kashmir University Botanical Garden, India. The landrace samples are collected during exploration programmes duly permitted and approved by Centre for Biodiversity and Taxonomy, University of Kashmir, and samples are documented properly. All the guidelines were followed as per the University research ethics for collection, characterization and documentation of landraces or germplasm accessions.

### Funding

This study did not receive any type of grant from a funding agency in the public, commercial, or not-for-profit sections.

## CRediT authorship contribution statement

**Shabeer Ahmad Dar:** Writing – original draft, Resources. **Ishfaq Shafi Khan:** Formal analysis, Data curation. **Gousia Majeed:** Conceptualization. **Sumira Tyub:** Software, Investigation, Formal analysis. **Irshad Ahmad Nawchoo:** Writing – review & editing, Supervision. **Azra Nahaid Kamili:** Writing – review & editing, Supervision, Project administration.

## Declaration of competing interest

The authors declared that they have no conflict of interest related to the publication of this article.

## Data Availability

Data will be made available on request.
